# Impact of COVID-19 on family planning

**DOI:** 10.18332/ejm/137484

**Published:** 2021-06-28

**Authors:** Bakhtawar M. H. Khowaja, Quratulain Shalwani

**Affiliations:** 1 Department of Obstetrics and Gynecology, Medical College, Aga Khan University, Karachi, Pakistan; 2 Institute of Physical Therapy and Rehabilitation, South City Hospital, Karachi, Pakistan

**Keywords:** Pakistan, family planning, COVID-19, LMICS

**Dear Editor,**

The COVID-19 impact on antenatal care of women has been well-explained by Ncube^1^ in her article ‘COVID-19 and antenatal care: An update’. And so, we would like to highlight the effect of COVID-19 pandemic on another aspect of maternity care services which is ‘family planning’.

The pandemic has upset the health systems of all countries worldwide and some of the countries with resilient health systems such as those in Europe and North America have contracted due to the impact of this cruel pandemic^2^. The emphasis is now on the low-middle-income countries (LMICs) to see how they fare in the control of the COVID-19 pandemic. The health systems of these countries are considered fragile and the added burden of a pandemic makes it difficult to confirm health services in these countries^3^. These countries struggle to handle the health burden due to pandemic related emergencies, and, in this struggle, routine essential healthcare services have been neglected^2^.

A UNFPA report determined that due to neglected health services in LMICs, about 47 million women in these countries will not be able to utilize contraception and this would result in 7 million unintended pregnancies^4^. The COVID-19 pandemic has further weakened the health systems of LMICs which are performing at suboptimal level due to limitations of skilled health workforce, logistics issues and organizational issues^3^. The initial efforts taken by all the countries to halt the epidemic of this infection included lockdown within their cities, regions and neighboring borders^4^. The little access to routine healthcare services due to lockdown and suspended transportation affected healthcare utilization by their citizens^5^.

The strategies to cease the spread of infection among the population have also disturbed supply chain processes further leading to non-accessibility of commodities and supplies for family planning^6^. The pandemic has negatively influenced family planning services due to significant reduction in human resources especially at primary health facilities^6^. The human resources, personal protective equipment and all other essential health facilities were diverted to the prevention of COVID-19 infection^4^. Limited resources and fear of spread of infection have reduced facilities for family planning in the private sector^5^.

Pakistan is on the list of LMICs and the health system of Pakistan, especially primary healthcare, is facing several challenges^7^. The Pakistan Demographics and Health Survey (2017–2018) demonstrates that the unmet need for contraception is high in Pakistan^7.^ COVID-19 has aggravated the situation by decreasing access to family planning services^6^. The health system of Pakistan should be restructured and reorganized to provide efficient health services delivery in these disrupted situations. The health system needs to be responsive and should focus on the comprehensive health needs of people rather than focusing on prevention of infection specifically. This would help in reduction of morbidities and mortalities due to various health conditions other than the pandemic.

A health system response for family planning services during the pandemic is very important in Pakistan to avoid unwanted pregnancies and prevent additional mortality and morbidity of women. To do so, tele-health and tele-clinic can be opted as an alternative strategy for family planning services. The health sector should work on providing knowledge to community health workers and community people about the use of tele-health services through different platforms to remove barriers for family planning services.
